# Long-Term Differential Effects of Gastric Bypass and Sleeve Gastrectomy on Bone Mineral Density

**DOI:** 10.1210/jendso/bvae111

**Published:** 2024-06-27

**Authors:** Pierre-Emmanuel Cailleaux, Agnès Ostertag, Didier Albert Haguenauer, Séverine Ledoux, Martine Cohen-Solal

**Affiliations:** Service de gériatrie aiguë, Hôpital Louis-Mourier, Assistance Publique—Hôpitaux de Paris, F-92700 Colombes, France; Inserm Bioscar, Université Paris Cité, 75010 Paris, France; Inserm Bioscar, Université Paris Cité, 75010 Paris, France; Service de gériatrie aiguë, Hôpital Louis-Mourier, Assistance Publique—Hôpitaux de Paris, F-92700 Colombes, France; Service des Explorations Fonctionnelles, Centre intégré de prise en charge de l’obésité (CINFO), Hôpital Louis-Mourier APHP.Nord, Colombes & Université Paris Cité, 92700 Colombes, France; Inserm Bioscar, Université Paris Cité, 75010 Paris, France

**Keywords:** sleeve gastrectomy, gastric bypass, bone mineral density, biomarkers, vascular calcification

## Abstract

**Context:**

The association of obesity with bone fragility fractures is complex and non-linear. Despite good efficacy on weight loss, bariatric surgery (BS) is also associated with bone loss. However, we lack information on risk factors of the long-term deleterious effects of BS on the skeleton.

**Objective:**

We aimed to assess the factors associated with low bone mineral density (BMD) performed a long time after Roux-en-Y gastric bypass (RYGB) or sleeve gastrectomy (SG).

**Methods:**

This cross-sectional study involved patients at a long distance from their BS that underwent dual-energy x-ray absorptiometry (DXA) with biological factors (vitamins, micronutrients, bone and inflammation biomarkers). Simple and multiple linear models (stepwise and parsimony approach) were developed.

**Results:**

A total of 131 patients (91 RYGB, 40 SG) underwent DXA (51.8 ± 11.08 years, 87.8% women). At a mean of 6.8 ± 3.7 years after surgery, the mean weight loss was –28.6 ± 9.6%, and only 6 patients (5.7%) had a *T*-score less than or equal to ­2.5. On univariate analysis, BMD was lower in the RYGB than in the SG group (*P* < .001) at all sites, despite similar fat and fat-free mass and weight loss. Serum parathyroid hormone and phosphate levels were higher in RYGB than SG patients. A total of 10.1% of patients showed vascular calcifications. On multivariable analysis, BMD remained different between surgery groups after adjustment for age, body mass index, ethnicity, and sex. The model-adjusted *R*^2^ values were 0.451 for the total hip; 0.462 the femoral neck, and 0.191 the lumbar spine for the inflammation model; 0.458, 0.462, and 0.254, respectively, for the bone marker model; and 0.372, 0.396, and 0.142 for the vitamin model. Serum zinc, ferritin, and uric acid levels were the markers associated with BMD to a low extent.

**Conclusion:**

BMD differed depending on the BS procedure. A few biological markers may be associated weakly with BMD well after the surgery.

Osteoporosis is a silent disease related to bone loss and is associated with risk of fragility fractures. The main risk factors are age and hormonal status [1], although during the last decade, obesity has been considered as a cause of fragility fractures. Obesity, defined by the World Health Organization as a high body mass index (BMI), is increasing worldwide, now reaching a prevalence of 10% to 20% for men and 15% to 25% for women in Europe [2].

Bariatric surgery (BS) is recommended for managing stage 3 or stage 2 obesity (BMI > 40 and > 35, respectively) with comorbidities [3]. Thus, the number of BS surgical procedures has markedly increased, with 604 223 primary interventions performed worldwide in 2021 [4]. Laparoscopic vertical sleeve gastrectomy (SG) and laparoscopic Roux-en-Y gastric bypass (RYGB) represent the majority of BS procedures. Long-term complications differ between the procedures because SG consists of 80% stomach removal, whereas RYGB bypasses the duodenum and the proximal jejunum. RYGB allows for 30% sustainable weight loss, and SG has higher interindividual variability [5]. In addition, RYGB is considered a malabsorptive procedure, more prone to nutritional issues than SG, including calcium deficiency and vitamin D insufficiency.

High BMI remains a protective factor against fracture risk, as people with obesity experience fewer osteoporotic fractures. In the National Health and Nutrition Examination Survey (NHANES) cohort, age-standardized incidence for hip fracture was 50 per 1000 patients in obese women older than 65 years vs 96 and 131 in normal and underweight women, respectively [6]. However, assessing fracture risk is more complex than in postmenopausal women, as the relationship between BMI and fracture risk is not linear. Osteoporotic fractures in obese patients seem to occur at specific sites. These osteoporotic fracture sites could be the ankle [7] and the vertebra [8]. As such, obesity is associated with a high bone mineral density (BMD) [9, 10] and a maintained bone microarchitecture and strength [11]. After surgery, rapid weight loss is also accompanied by muscle loss and bone loss. Fracture risk is increased after BS as compared with conservative treatment, especially in RYGB [12, 13]. Patients who underwent BS seemed more likely to have fractures than patients with obesity, as reported in a nested case-control study over 10 years in Quebec [14]. Fracture risk was increased 2.3-fold in a cohort of patients undergoing RYGB (94%) in 1985 to 2004 in Minnesota [15]. Nonetheless, when SG is taken into account, the effect of BS on fracture risk is less reported [16]. The specific role of RYGB in major osteoporotic fracture incidence (hazard ratio 1.70; 95% CI, 1.46-1.98) was also reported in a French National Inpatient database analysis between 2008 and 2018 [17]. In both surgical procedures, BMD rapidly decreases depending on the extent of weight loss, as shown in longitudinal studies [11, 18], with a prevalence of BMD osteoporosis (T-score ≤ −2.5) estimated at 27% at 10 years for RYGB [19]. BMD decreases linearly at all sites with RYGB but at the femoral neck only with SG [20]. In RYGB, bone microarchitecture degradation continues within 2 years despite weight stabilization [21, 22]. However, the factors associated with bone loss are only partially characterized [23].

Yet, the European Calcified Tissue Society position statement and the French guidelines recommend performing dual-energy x-ray absorptiometry (DXA) twice (at baseline and ∼2 years after surgery) after BS, in patients older than 50 years [24, 25]. For metabolic and nutritional parameters, the International Federation for the Surgery of Obesity guidelines recommend performing only at baseline an extensive set of assessments including testing for calcium metabolism biomarkers [3]. Only a few studies have provided long-term DXA evaluations with both BS procedures [19, 21], and the bone and nutritional parameters associated with BMD at a long distance from surgery still remain poorly reported.

In this study, we aimed to investigate the factors potentially associated with BMD (vitamins, micronutrients, metabolic and inflammatory biomarkers) in a cross-sectional study of patients who underwent their BS a long time ago (≥2 years from surgery).

## Materials and Methods

We analyzed the data for a monocenter population-based cohort of patients referred for BS in a University Hospital of Paris area (Louis-Mourier Hospital, AP-HP, Colombes). The cohort study aimed to prospectively identify the consequences of BS in people with obesity and notably nutritional complications with repeated systematic assessments before and after surgery. Although the prescription of DXA was systematic before and every 3 years after surgery, attendance remained low.

For this cross-sectional study, we assessed a subset of patients who underwent postoperative BMD measurement at our university hospital between 2020 and 2021. Inclusion criteria were as follows: all patients followed in the obesity cohort who underwent surgery 2 or more years before DXA assessment on site, and having clinical and biological data performed at our center before and close to (<12 months) DXA assessment. We excluded only patients who underwent surgery in the last 2 years, those who refused the use of their data, and those who did not perform their complete follow-up on site (DXA and biology assessments) in 2020 to 2021. The study protocol was approved by the institutional review board of the French Institute of Medical Research and Health–Inserm (2023-CEEI/IRB00003888, IORG0003254, FWA00005831no 23-1038). Patients were included if they did not express their opposition to the study after they were given specific information.

### Clinical and Biological Parameters

Clinical data recorded included demographics (age, sex, and ethnicity), anthropometrics (height, weight), comorbidities, and medications. We were unable to analyze fracture data because the collection of data on fracture history was incomplete.

Routine biological parameters included the levels of calcium metabolism factors (serum and 24-hour urine calcium, serum phosphate, serum magnesium, 25 (OH) vitamin D, intact parathyroid hormone [PTH], creatinine, and total alkaline phosphatases [TALPs]); other micronutrients (iron, transferrin saturation, ferritin, zinc, and selenium); and vitamins (serum A, B1, B3, B6, B9, B12, E). Nutritional, inflammatory, or metabolic factors measured included serum levels of hemoglobin, homocysteine, albumin, prealbumin, fasting blood glucose and insulin, triglycerides, cholesterol, uric acid, and C-reactive protein (CRP). All factors were assessed during fasting, using routine techniques previously described [26].

### Bone Mineral Density Measurement

DXA assessment included BMD measurement, systematic vertebral morphometry (for vascular calcification quantification), and whole-body composition assessment (body lean mass and fat mass and their distribution: visceral and subcutaneous adipose tissue). BMD was measured in the same center by using a single iDXA device (GE-Lunar Medical Systems Lunar) available since late 2019. BMD was measured at the femoral neck, total hip, and lumbar spine. We assessed the areal BMD (g/cm²), Z-scores, and T-scores using the NHANES reference population. The stability of the measurements was checked every day by using a phantom for calibration. The mean coefficient of variation for measurement for the lumbar spine was 0.41%; total hip, 0.53%; and femoral neck, 1.36%. Because the previous BMD examinations were not available for all patients, we were unable to provide data for bone loss. Vascular calcification was assessed during vertebral morphometry assessment by using the Kauppila score (0-24). A trained operator scored the 4 lumbar vertebra levels for all patients included.

### Statistical Analysis

Descriptive data are presented as mean ± SD and number (percentage) for categorical data. Comparisons between groups involved the chi-square test for categorical variables (or Fisher exact test). Quantitative variables were compared with the *t* test or analysis of variance depending on the number of variables. In case of significance of the analysis of variance, a post hoc Tukey test was conducted. In case of heteroscedasticity, we used a Mann-Whitney *U* test (2 comparisons) or Kruskal-Wallis test (>2 comparisons). Shapiro tests assessing the normality and homogeneity of variance were checked with Bartlett tests.

Comparisons between 2 continuous variables involved simple linear regression. Variables with *P* less than .2 on univariate analysis were included in the parsimony model, including variables forced in the model for which an association was already established (age, sex, ethnicity, and BMI). We developed 3 multivariable linear models. Hence, in the univariate analysis, the different factors were divided as follows:

- inflammation biomarkers (levels of CRP, serum homocysteine, homocysteine/creatinine, and ferritin; kaolin cephalin coagulation time; prothrombin rate; fibrinogen level);
- mineral and bone biomarkers (levels of serum calcium and phosphate, intact PTH, thyrotropin (TRH), serum magnesium, TALPs, iron, creatinine, urate, 24-hour urinary calcium, 24-hour urinary phosphate);
- for other vitamins and micronutrients (levels of vitamin A, B1, PP, B6, B9, B12, D, E, zinc, selenium).

We then developed multiple linear parsimony models with stepwise selection for adjusted variables. Age, sex, BMI, and the type of surgery were systematically included in the 3 multivariable analyses. Time from surgery variable was eliminated from the multivariable analysis because of a correlation with the age of patients, a stronger factor. For comparisons between BMD sites, we excluded all patients for whom data were not available at each DXA site. A *P* value less than .05 was considered statistically significant. All tests were 2-sided. Statistical analysis involved using *R* v4.1.2 ([2021-11-01], The R Foundation for Statistical Computing) and the following packages: car (3.0-12) and Hmisc (4.6-0).

## Results

### Patient Characteristics

More than 2 years after surgery, data for 131 patients who underwent both DXA body scan and biology assessments were available for analysis: A total of 91 (69.5%) had RYGB and 40 (30.5%) SG. At the time of inclusion, the mean time from BS was 6.8 ± 3.7 years (Table 1). Participants were mostly women (87.8%), and the mean age was 51.8 ± 11.1 years, mean BMI 32.1 ± 5.71 and weight loss −28.6 ± 9.6% as compared with their preoperative weight.

**Table 1.** Clinical and biological characteristics of patients by type of bariatric surgery: Roux-en-Y gastric bypass or sleeve gastrectomy

	Total	RYGB	SG	*P*
		n = 91	n = 40	
Age, y	51.8 ± 11.08	54.2 ± 10.35	46.4 ± 10.93	<.001
Sex, female	115 (87.8%)	79 (86.8%)	36 (90%)	.775
Black ethnicity, %	24 (18.32%)	18 (19.7%)	6 (15.0%)	.515
Preoperative BMI	45 ± 6.06	45.7 ± 6.43	43.5 ± 4.87	.059
Current BMI	32.1 ± 5.71	32.1 ± 5.68	32.2 ± 5.87	.893
Time from surgery, mo	81.6 ± 44.2	96.1 ± 44.21	48.5 ± 19.74	<.001*^a^*
Relative weight loss, %	−28.6 ± 9.60	−29.6 ± 9.50	−26.2 ± 9.49	.056
Proton pump inhibitors	32 (24.4 %)	17 (18.7%)	15 (37.5%)	.021
Multivitamin supplements	114 (0.87)	85 (93.4%)	29 (72.5%)	<.001
Iron supplements	27 (20.6 %)	20 (22%)	7 (17.5%)	.56
Vitamin B_12_ supplements	23 (0.166)	20 (22%)	3 (7.5%)	.088
Calcium supplements	28 (21.4 %)	25 (27.5%)	3 (7.5%)	.01
Vitamin D supplements	66 (50.4 %)	52 (57.1%)	14 (35%)	.02
**Biological factors**				
C-reactive protein, mg/L	2.37 ± 2.88	2.14 ± 2.65	2.88 ± 3.32	.005*^a^*
Fibrinogen, g/L	3.48 ± .69	3.56 ± 0.7	3.31 ± 0.64	.057
Homocysteine, µmol/L	10.68 ± 4.45	11.4 ± 4.92	8.98 ± 2.31	.001*^a^*
Ferritin, µg/L	78.18 ± 80.74	70.14 ± 78.32	96.48 ± 84.13	.086
Serum albumin, g/L	39.00 ± 3.25	38.90 ± 2.93	39.10 ± 3.93	.379
Serum calcium, mmol/L	2.29 ± 0.1	2.3 ± 0.1	2.28 ± 0.09	.276
Serum phosphates, mmol/L	1.27 ± 0.15	1.3 ± 0.14	1.19 ± 0.15	<.001
Serum magnesium, mmol/L	0.83 ± 0.08	0.83 ± 0.07	0.82 ± 0.09	.445
Zinc, µmol/L	10.95 ± 1.64	10.9 ± 1.52	11.08 ± 1.92	.613
Selenium, µmol/L	1.25 ± 0.37	1.26 ± 0.41	1.23 ± 0.25	.677
Iron, µmol/L	15.15 ± 5.49	14.8 ± 5.73	15.95 ± 4.88	.272
25 (OH) vitamin D, nmol/L	66.08 ± 23.26	65.92 ± 22.9	66.43 ± 24.34	.908
Serum creatinine, µmol/L	64.17 ± 15.58	65.35 ± 17.47	61.48 ± 9.69	.260*^a^*
24-h calcium, mmol/24 h	3.35 ± 1.55	3.33 ± 1.51	3.41 ± 1.67	.910
Uric acid, µmol/L	252.6 ± 63.4	256.3 ± 65.4	244.1 ± 60.6	.323
TSH, mUI/L	2.03 ± 1.40	2.01 ± 1.04	2.1 ± 2.0	.454*^a^*
Parathyroid hormone, pg/mL	48.54 ± 23.06	52.95 ± 24.89	38.92 ± 14.59	.001*^a^*
Total alkaline phosphatase, UI/L	83.09 ± 26.37	89.31 ± 26.8	68.95 ± 19.08	<.001
Serum potassium, mmol/L	4.34 ± 0.36	4.36 ± 0.38	4.29 ± 0.32	.320
Serum bicarbonates, mmol/L	29.40 ± 2.26	29.43 ± 2.27	29.33 ± 2.25	.802

Data are mean ± SD unless otherwise indicated.

Abbreviations: BMI, body mass index; RYGB, Roux-en-Y gastric bypass; SG, sleeve gastrectomy; TSH, thyreostimulin.

^*a*^Nonparametric comparison test (Mann-Whitney for 2 variables, Kruskal-Wallis otherwise).

Participants differed by BS surgery type in age and time from surgery (see Table 1). SG patients were younger than RYBG patients and were assessed at less time from the procedure because SG is a more recent technique. They did not differ in weight loss or BMI long after the procedure (*P* = .056 and *P* = .893, respectively). Body composition was similar with both procedures for lean mass and subcutaneous and visceral fat mass (Supplementary Table SI [27]). Overall, 16.8% of the patients had type 2 diabetes mellitus, with a similar prevalence with both procedures (Supplementary Table SII [27]). Total cholesterol and low-density lipoprotein levels were lower for RYGB than SG patients (*P* < .001), with no other difference in lipid levels. Prescriptions for proton pump inhibitors were more frequent for SG than RYGB patients, with less elevation in folic acid level (serum vitamin B_9_) and no insufficiency because of less supplement prescription.

For serum inflammatory markers in the whole population, CRP, ferritin, and fibrinogen levels were above the normal range for 10 (7.6%), 2 (1.5%), and 21 (16.4%) patients, respectively (see Table 1). Despite similar BMI, SG patients had higher levels of CRP than RYGB patients (*P* = .005 Mann-Whitney *U* test). Serum calcium and phosphorus levels were within the normal range for all patients. PTH level was elevated (>65 pg/mL) in 22 patients (17.7%), with no alteration in serum calcium and phosphate levels and no renal impairment. PTH level was found to be elevated more in RYGB than SG patients (*P* = .001). Among patients with an elevated PTH level, 17 (77.3%) had vitamin D insufficiency (<75 nmol/mL) and 7 (31.8%) vitamin D deficiency (<50 nmol/L). Furthermore, TALP level was higher in RYGB than SG patients (*P* < .001), with no difference in aspartate transaminase and γ-glutamyltransferase content. Phosphate level was higher in RYGB than SG patients, with normal and similar vitamin D level and calcium serum and urine levels, despite a supposed difference in calcium and vitamin D intake: more prescriptions for supplements in RYGB than SG patients because of less absorption.

The mean serum vitamin levels were within the normal range in most of these patients with supplementation. For all patients, 23.7%, 6.9%, 26.5%, 6.1%, 6.1%, and 18.6% had serum vitamin levels below the laboratory threshold for vitamin A, B1, PP, B_6_, B_12_, and E, respectively. Overall, 80% of the population had zinc insufficiency and 5.7% selenium insufficiency.

### Dual-Energy X-Ray Absorptiometry Assessment (Univariate Analysis of Bone Mineral Density, Body Composition, and Vascular Calcification)

**Figure 1.**
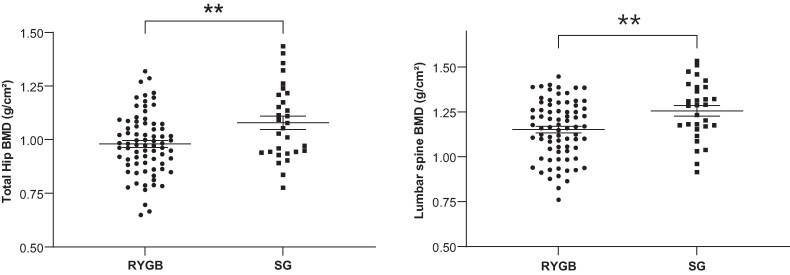
This figure displays univariate comparisons of bone mineral density (BMD in g/cm²) between Roux-en-Y gastric bypass (RYGB), and sleeve gastrectomy (SG), at 2 BMD measurement sites (total hip and lumbar spine). BMD is significantly lower in RYGB at both sites.

**Figure 2.**
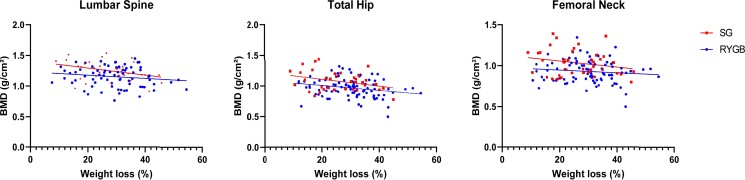
This figure displays 3 scatter plots for each bone mineral density (BMD in g/cm²) measurement site (lumbar spine, total hip, and femoral neck) according to the extent of weight loss (in %).

In the whole population, only 6 (5.7%) patients had osteoporosis according to the World Health Organization definition. As expected, BMD differed by sex (greater BMD in men than women) and age. Without adjustment, BMD was greater in SG than RYGB patients regardless of measurement site: lumbar spine, total hip, or femoral neck (Fig. 1 and Supplementary Table SI [27]). BMD was also greater with high BMI, but only at the hip (total hip *P* < .010; femoral neck *P* = .020). BMD was also associated with the extent of weight loss in the whole population at each site: lumbar spine (*P* = .016; *R*^2^ = 0.053), total hip (*P* < .001; *R*^2^ = 0.1), and femoral neck (*P* = .003; *R*^2^ = 0.04). This significant effect was present for both BS types at all sites, with a more pronounced effect in SG than RYGB patients, unlike body fat mass and lean mass, which remained similar between the surgery types (see Supplementary Table SI [27]). Fig. 2 displays the crude relationship between BMD and relative weight loss for each surgery type. The relation was significant and negative at the total hip for both procedures (RYGB: *P* = .022; *R*^2^ = 0.058; SG: *P* = .046; *R*^2^ = 0.109) and at the lumbar spine for SG only (*P* = .047; *R*^2^ = 0.125).

We further assessed the presence and extent of vascular calcification on systematic lateral spine x-ray morphometry. Fifteen patients (10.1%) had vascular calcification (Supplementary Fig. S1 [27]). The calcification level did not differ between type of surgery (*P* = .927) and according to BMI (*P* = .680).

### Multivariable Analysis of Biological Determinants of Bone Mineral Density

Multivariable analysis involved only patients with complete data for BMD at all sites. Parameters retained after univariate analysis were prothrombin rate and levels of ferritin, CRP, and homocysteine/creatinine for the inflammation biomarker model. Bone biomarkers included were serum phosphate, PTH, TALP, thyreostimulin (TSH), serum magnesium, and uric acid levels. Finally, vitamin A, B_1_, B_9_, D, and E and zinc levels were included in the multivariable model of vitamins and micronutrients.

In all models, the association with BMD was stronger (higher adjusted *R*^2^) at the total hip and femoral neck than lumbar spine. For the inflammation model, the adjusted *R*^2^ was 0.451 for the total hip, 0.462 the femoral neck, and 0.191 the lumbar spine. For the mineral and bone markers model, the adjusted *R*^2^ values were 0.458, 0.462, and 0.254, respectively, and for the vitamins and micronutrients model, 0.372, 0.396, and 0.142, respectively. BMD values remained higher at all BMD sites in SG patients, after adjustment for all batches.

Among vitamins and micronutrients, serum zinc was the only biological remaining factor. Serum zinc concentrations were positively associated with BMD (*P* = .0373) at the femoral neck to a low extent (Table 2). The same relationship was found when the zinc variable was dichotomized by biological threshold. Among the inflammatory markers, ferritin level was the only factor involved in the parsimony model at the total hip (data not shown) and the femoral neck, with a significant negative relationship (see Table 2). Uric acid level was the only significant factor negatively associated with both total hip and femoral neck BMD, with a slight but significant negative effect on BMD at the total hip (data not shown) and femoral neck (see Table 2) (estimate = −0.8; *P* value = .0284 and estimate = −0.5; *P*-value = .042, respectively). In contrast, none of the bone biomarkers from the 3 panels was included in the final models assessed.

**Table 2.** Final multivariable linear models for factors associated with bone mineral density at the femoral neck for the 3 batches

Vitamin micronutrients (n = 106)	Estimate	SE	*P*	Inflammation (n = 99)	Estimate	SE	*P*	Bone biomarkers (n = 98)	Estimate	SE	*P*
(Intercept)	810	128.7	<.001	(Intercept)	949	85	<.001	(Intercept)	916	81.9	<.001
Type of BS (ref = RYGB)	67.9	31.5	.0345	Type of BS (ref = RYGB)	81.3	27.6	.004	Type of BS (ref = RYGB)	66.7	26.6	.014
Sex (ref = women)	60.2	45.3	.1883	Sex (ref = women)	83.9	39.2	.035	Sex (ref = women)	125	43.2	.005
Age	−5.6	1.4	<.001	Age	−6.2	1.2	<.001	Age	−6.4	1.2	<.001
Black ethnicity (ref = White)	49.1	32.8	.1381	Black ethnicity (ref = White)	77.5	28.9	<.009	Black ethnicity (ref = White)	84.5	29.9	.019
BMI	8.4	2.6	.0023	BMI	11.2	2.2	<.001	BMI	14.4	2.6	<.001
log(vitamin A)	−87.5	55.4	.1183	log(ferritin)	−2.11	10.2	.041	Uric acid	−0.5	0.2	.042
Serum zinc	17.4	8.2	.0373								

Abbreviations: BMI, body mass index; BS, bariatric surgery; ref, reference; RYGB, Roux-en-Y gastric bypass.

## Discussion

In this well-characterized population of people at a mean of 6.8 years after surgery, we found that BMD remained lower with RYGB than SG after adjustment for age, sex, ethnicity, and BMI. We also shown that, to a low extent, the biological markers ferritin, zinc, and uric acid levels were associated with BMD after adjustment. Well after surgery, the association was stronger with BMD at the total hip and femoral neck than the lumbar spine.

Decreased areal BMD has been previously described in several studies with small populations. Most of them assessed longitudinal bone loss within 2 years after surgery [20, 28-31]. Bone loss occurs at all sites for both procedures. However, the total hip seems the most relevant DXA measurement site after BS among RYGB patients according to a recent meta-analysis [32]. Early bone loss already differs by type of surgical procedure [20], which suggests both shared and specific risk factors for low BMD. This early bone loss occurs according to the extent of weight loss at the hip at 1 year after RYGB [18] and at 2 years after SG [33]. In SG patients, hip BMD changes were also associated with changes in lean mass but not body weight [20]. Nonetheless, weight stabilization is expected in both procedures, as confirmed in a 5-year follow-up study [34]. However, despite weight stabilization, bone microarchitecture, studied with HR-pQCT (high-resolution peripheral quantified computed tomography), is deteriorated at 2 years after RYGB, despite maintenance of bone biomarkers [22]. In addition, this cortical bone loss observed in women after RYGB seems to preferentially affect the mid-cortical and periosteal layers [35] rather than the classic endosteal trabecularization seen after hormonal deprivation [36]. The hypothesis of the involvement of physical function has been drawn. However, this effect remains unclear: In 21 RYGB patients, weight variation but not daily gravitational loading predicted decreased BMD (total hip and femoral neck) [37]. Thus, RYGB shows differential BMD, bone biomarkers, bone microarchitecture, and fracture risk as compared with SG, since early after surgery, with comparable weight loss and weight stabilization. Our study confirmed that there is still a difference in BMD in a cross-sectional approach at a long distance from surgery.

No bone biomarkers remained as associated factors with BMD in our multivariable models, suggesting their relatively low effect on BMD in our BS patients. However, in the univariate analysis, we found lower BMD and higher PTH and TALP levels after RYGB than SG. Hence, the persistent difference between BS procedure could be explained by the persistent malabsorption in RYGB. PTH is significantly higher in RYGB, although 25 (OH) vitamin D levels are equal, and RYGB were more frequently supplemented (see Table 1) with a similar process in dose delivery between groups. Thus, the role of vitamin D supplementation has not been fully elucidated and other causes of PTH elevation should be discussed. We searched for a higher prevalence of a cause of secondary hyperparathyroidism between groups: in creatinine levels (*P* = .260), 24-hour urinary calcium levels (*P* = .910), and in thiazide diuretics prescription (*P* = 1). An abnormal prevalence of 83.7% of hyperparathyroidism in the sixth year after RYGB, also partially explained by vitamin D, has been recently described in RYGB patients [38]. As the absorption of 25 (OH) vitamin D and calcium are decreased after RYGB [39], we could hypothesize to explain the higher PTH levels in RYGB, that in our work, with higher amounts of vitamin D, we reached a similar normal level in serum and 24-hour urinary calcium, suggesting a persistent malabsorption of vitamin D. The increase in TALPs is cohesive with these results, as increased bone-specific ALP content was described after RYGB [20], which suggests increased bone turnover level [24]. Phosphate level, although in the normal range, was higher after RYGB than SG. No renal impairment or reduced PTH level could explain this singularity, except that PTH could be elevated in response to elevated phosphate, for its phosphaturic effect, but we lack information to conclude. This result remains, however, poorly reported. In a study of BS patients at a mean of 16.9 ± 8.1 years follow-up, phosphate levels were stable, with no difference between procedures [40]. Another approach to explain these differences in BMD and biomarkers with the malabsorptive hypothesis was to investigate loss of alkali related to malabsorption diarrhea. Markers of subclinical acidosis are low serum potassium or bicarbonate and low urinary citrate [41]. This acidosis involves a buffering by bone tissue and an increased bone dissolution. Lower serum bicarbonate levels are positively related to lower BMD [42]. In our study, both serum potassium and bicarbonate were not low and were similar between the groups at the assessment point (*P* = .320 and .802, respectively).

Ferritin was also found to be associated with BMD in our population, to a low extent. Ferritin is a protein that reflects iron stock. Both surgical procedures lower iron levels [43], suggesting that in our population with normal ferritin levels, patients underwent adapted supplementation. Iron overload, such as in hemochromatosis, is related to bone architecture impairment, as reported in a recent HR-pQCT analysis [44] and expose patients to cirrhosis and hypogonadism, both of which are related to low BMD. Ferritin is also related to inflammation through macrophages and stores iron in the liver in specialized macrophage cells. As obesity is associated both with fatty liver disease and low-grade inflammation, we could hypothesize that high ferritin levels also reflect low BMD to a low extent. Based on data from NHANES, increases in serum ferritin imply a higher risk of low lumbar spine BMD [45]. Interestingly, fibroblast growth factor-23 concentration that drives phosphate metabolism is related to iron and obesity status, suggesting the involvement of fibroblast growth factor-23 as a potential third part [46].

We also found a significant negative relationship of low extent between uric acid and BMD. Obesity is often accompanied by hyperuricemia, as the adipose tissue produces and secretes uric acid through xanthine oxidoreductase. Uric acid seems to be an indicator of metabolic outcomes, such as metabolic syndrome features [47]. Data on the direction relationship between BMD and uric acid are diverse according to the population studied [48, 49] but with no causal relationship identified between uric acid levels and BMD [50]. Recent findings have shown that lumbar spine BMD was associated with hyperuricemia in obese men but not in women [51]. However, as our results were adjusted for BMI and sex, we suggest another hypothesis to explain the effect of uric acid on BMD. Uric acid was not related to the presence of type 2 diabetes mellitus, glycated hemoglobin A_1c_, fibrinogene, and CRP in our study. However, it was related to triglycerides (*P* = .028; *R*^2^ = 0.0375) and γ-glutamyltransferase (*P* < .001; *R*^2^ = 0.089) to a low extent. We can hypothesize that the effect of uric acid could be partially mediated by a liver dysfunction related to obesity, such as nonalcoholic fatty liver disease.

We also studied vitamin levels that did not affect BMD in patients undergoing BS. We found a positive association between zinc level and DXA at the femoral neck and total hip. Zinc deficiency has been reported in patients after BS but was not found related to BMD [52]. Otherwise, some preclinical studies suggest a potential role of zinc in the downregulation of the RANK/RANK-l signaling pathway [53]. Further research on humans is needed to determine a potential effect of zinc supplementation on bone resorption.

One of this study's strengths is the long-distance assessment of well-characterized patients. We used an original approach in studying the association between BMD and vitamin levels, vascular calcification level, and micronutrient levels. Our multivariable models provided appropriate adjustments to compare the 2 surgery types. However, this study suffers from limitations. The main limitation remains the cross-sectional design, with the difficulty in obtaining serial DXA examinations, which limited conclusions for the evolution of BMD over time. Differences in factor associations may have been preexisting. Lack of complete information on fractures, menopause status, and bone-related medications also limited the interpretation. The selection and allocation of patients and factors could raise some limitations and selection bias. In addition, statistical adjustment mitigates but does not eliminate confounding arising from imbalances in the study groups. Moreover, factors identified in the multivariable analysis remained poorly supported by the present data.

## Conclusion

In a cross-sectional analysis at a long distance from BS, BMD still differed at all sites by surgical procedure. Some arguments suggest a persistent malabsorptive effect after RYGB. Serum zinc was positively associated with BMD, while ferritin and uric acid levels were negatively associated with BMD on multivariable analysis, all to a low extent, suggesting further explorations. Ongoing lifetime DXA screening will help in understanding the effect of BS on bone.

## Data Availability

Some or all data sets generated during and/or analyzed during the current study are not publicly available but are available from the corresponding author on reasonable request.

## Abbreviations

BMD: bone mineral density
BMI: body mass index
BS: bariatric surgery
CRP: C-reactive protein
DXA: dual-energy x-ray absorptiometry
HR-pQCT: high-resolution peripheral quantified computed tomography
NHANES: National Health and Nutrition Examination Survey
PTH: parathyroid hormone
RYGB: Roux-en-Y gastric bypass
SG: sleeve gastrectomy
TALPs: total alkaline phosphatases
TRH: thyrotropin
TSH: thyreostimulin
